# Quantum Yield and Fatty Acid Profile Variations With Nutritional Mode During Microalgae Cultivation

**DOI:** 10.3389/fbioe.2018.00111

**Published:** 2018-09-25

**Authors:** M. V. Rohit, S. Venkata Mohan

**Affiliations:** ^1^Bioengineering and Environmental Sciences Lab, EEFF Centre, CSIR-Indian Institute of Chemical Technology (CSIR-IICT), Hyderabad, India; ^2^Academy for Scientific and Industrial Research (AcSIR), Ghaziabad, India

**Keywords:** mixotrophic, microalgae, photosynthetic efficiency, polyunsaturated fatty acids (PUFA), lipids

## Abstract

Microalgae are gaining commercial interests in the areas food, feed and biofuel sector. They have intrinsic ability to harness energy from sunlight and photosynthetically valorize CO_2_ into various bio-based products viz., triacylglycerols (TAGs), mono/poly-unsaturated fatty acids (MUFA, PUFA), pigments etc. Microalgae have adapted to grow in various nutritional environments due to their metabolic versatility and resilience. Strategic evaluation of newly isolated strain *Chlorella* sp. from a residential lake was performed. The strain was investigated by varying the nutritional modes to gain insights into biomass and fatty acids production. Maximum biomass (3.59 g/L) was observed in mixotrophic condition followed by heterotrophic (1.58 g/L) and autotrophic condition (0.59 g/L). The maximum lipid yield (670 mg/g DCW) was observed in mixotrophic condition whereas maximum total lipid content (36%) was observed in heterotrophic condition. Significant correlation was noticed between fluorescence parameters measured by OJIP and non-photochemical quenching (NPQ) with the function of nutritional mode variations. Autotrophic condition showed higher photosynthetic activity which was well correlated with high fluorescence intensity as represented by OJIP, NPQ1, and NPQ2 curves. Good balance of saturated fatty acids (SFA) and unsaturated fatty acids was observed in autotrophic mode, whereas polyunsaturated fatty acids (PUFA) and mono unsaturated fatty acid (MUFA) content were relatively higher in mixotrophic and heterotrophic conditions.

## Introduction

Microalgae produce commercially important biomolecules viz. proteins, carbohydrates, lipids, vitamins, pigments, and other biologically active compounds having direct applications in food and health, biofuel, pharmaceutical and cosmetic sectors (Mayfield and Golden, 2015). Algal cultivation offers a sustainable strategy toward biofuel production while leaving a milder carbon footprint to the environment. Autotrophic mode enables sequestration of CO_2_ from atmosphere and can be integrated with industrial flue gases but with slower growth rates (Jia et al., 2015; Yen et al., 2015). Heterotrophic cultivation replaces light requirement by organic carbon source and can assimilate complex carbon to produce high density biomass (Lowrey et al., 2015; Venkata Mohan et al., 2015). Mixotrophic growth is the sum of individual growths of autotrophic and heterotrophic modes and explores the advantages of both CO_2_ and organic carbon as energy source (Blanken et al., 2014; Chandra et al., 2014; Rohit and Venkata Mohan, 2016). Mixotrophic cultivation allows large volume applications such as high biomass productivity when coupled with wastewater treatment and nutrient recovery towards value addition. Nutrient stress in the form of nitrogen depletion is considered favorable for triacylglycerol (TAG) accumulation in microalgae apart from other influencing factors like light, pH, temperature, oxidative stress and salinity (Devi et al., 2013; Chiranjeevi and Venkata Mohan, 2016). Monitoring microalgae growth based on photosynthetic activity gives insights into the nanoscale architecture of photosystems and enhances understanding of the photo-regulatory reactions taking place in the algal cells. Pulse Amplitude Modulation (PAM) Fluorometry is increasingly used as a non-invasive and rapid technique to measure the variability of chlorophyll fluorescence and photosynthetic performance both in plants and microalgae (Juneau et al., 2005; Baker, 2008). The parameters that are generally used to measure the physiological state of the photosynthetic organisms are non-photochemical quenching (NPQ), quantum yield, photosynthetic efficiency (Fv/Fm), relative electron transport rate (rETR), and light saturation (Ek). In microalgae, exposure to photosynthetically supersaturating light triggers the activation of energy dissipating processes that lowers the yield of chlorophyll “a” fluorescence and thus is generally termed as NPQ process (Masojídek et al., 1999).

In this study, the isolated strain *Chlorella* sp. SVMBIOEN3 was evaluated for biomass growth and lipid production with effect of changes in nutritional mode. The influence of nitrogen limitation on lipid and fatty acid profiles was elucidated. The changes in nutritional mode resulted in significant variability of photosynthetic efficiency in terms of quantum yield and non photochemical quenching (NPQ). The concentrations of individual fatty acids showed varying saturation and unsaturation profiles. The study correlates nutritional modes influence on changes in biomass and fatty acid content during algal cultivation supported by fluorescence kinetics.

## Materials and methods

### PCR amplification and phylogenetic characterization of strain

*Chlorella* sp. SVMBIOEN3 was isolated from Nacharam Lake, Hyderabad according to Rohit and Venkata Mohan (2016). For strain identification, genomic DNA was extracted from the isolated strain using Plant DNA isolation kit (Macharey Nagel) according to the supplier's protocol. ITS regions were amplified by using the primers ITS1F “TCCGTAGGTGAACCTGCGG” and ITS4R “TCCTCCGCTTATTGATATGC” (White et al., 1990). A 25-μl reaction was setup by using 1 μl template with 0.5 μl each of forward and reverse primer, 12.5 μl of 2X red dye PCR master mix (Takara) and remaining with PCR grade water. DNA amplification was carried out in a thermal cycler (Eppendorf) with the following program: initial denaturation at 94°C for 3 min, followed by 30 cycles of denaturation for 1 min at 94°C. The annealing temperature used was 51°C for 1 min. Polymerization step was performed at 72°C for 1.5 min and final elongation at 72°C for 10 min. Amplified PCR products were tested on 1% agarose gel. The PCR products were sequenced at Bioserve sequencing facility, and closest relatives were identified by BLAST tool of NCBI (http://www.ncbi.nlm.nih.gov/). Partial ITS gene sequences was submitted to GenBank NCBI database, and the accession number KY350162 was assigned.

### Experimental methodology

**Figure 1:**
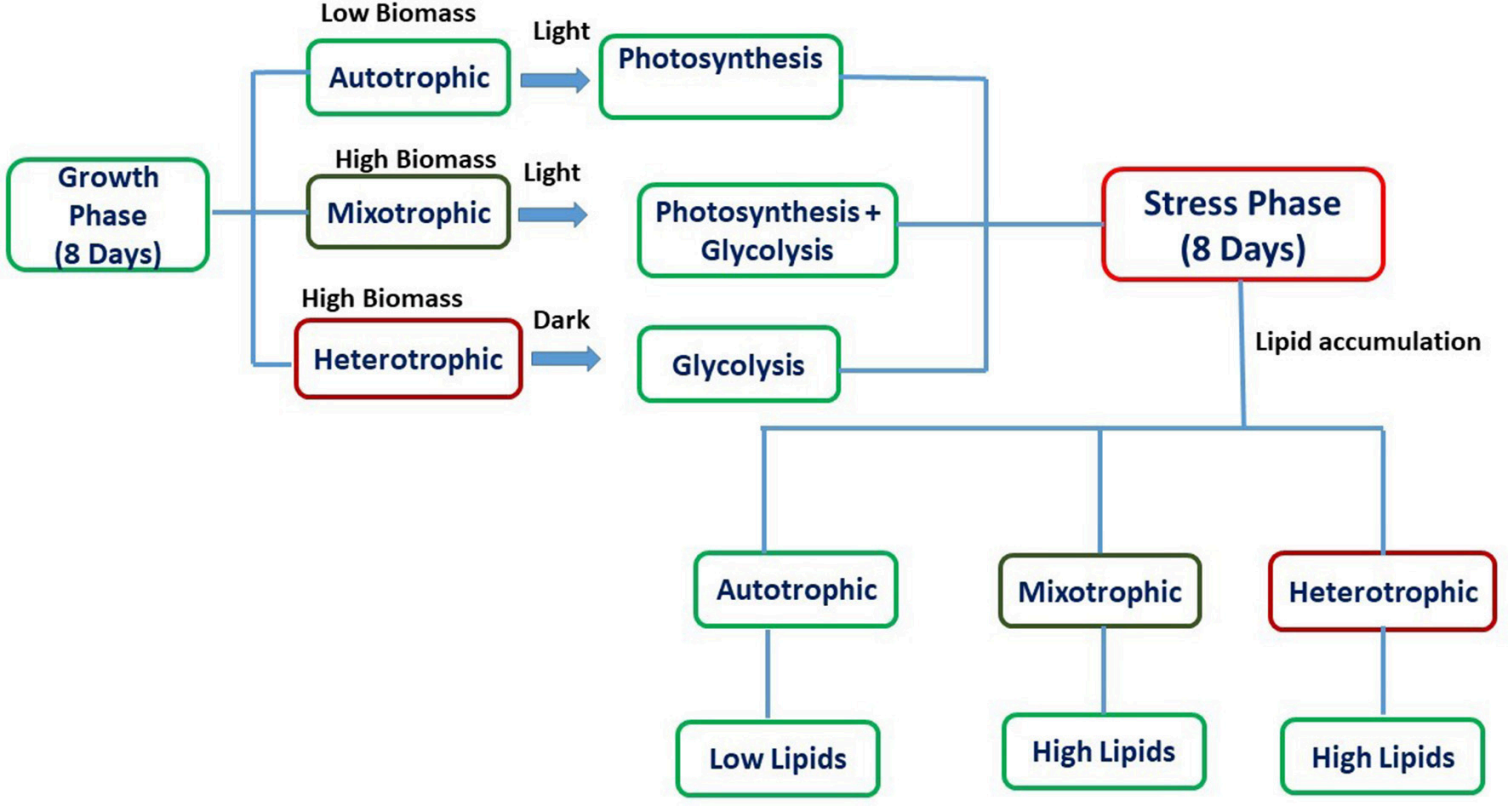
Schematic representation of biomass and lipid production in various nutritional modes.

Experiments were carried out for 16 days which was divided into two phases viz. growth phase (GP) and stress phase (SP). The retention time for growth phase (GP) and stress phase (SP) was 8 days each with temperature of 25°C and 7.1 pH in shaking incubator (120 rpm). The experiments were conducted in conical flasks with a total/working volume of 500/300 mL and exponentially growing *Chlorella* sp. SVMBIOEN3 cells from stock culture (10%) were inoculated into three flasks under sterile conditions. BG-11 medium was used for all the experiments. After 8 days of growth phase (GP), the culture was centrifuged and inoculated into sterilized BG-11 media without NaNO_3_ for maintenance of stress conditions and termed as SP. Glucose (7.5 g/L COD) was supplemented as organic carbon to BG-11 medium for mixotrophic and heterotrophic conditions. Atmospheric air served as inorganic carbon source for autotrophic experiments. Autotrophic and mixotrophic cultivations were provided with light intensity of 56 μE/m^2^/s using white fluorescent tube lights (Philips) with Light: Dark (12:12) cycle. The light intensity was measured using Extech LT-300 Light meter (Extech LT-300). Heterotrophic flasks were wrapped with aluminum foil to maintain dark conditions. Ampicillin (0.2 mg/mL) was added to avoid bacterial growth during algal cultivation. The schematic representation of the experiment with respect to nutritional modes is represented in Figure 1. All the experiments were performed in triplicates, and the standard deviation (SD) was represented in the form of error bars.

### Analysis

Biomass growth in terms of absorbance (750 nm) was determined using UV-Vis spectrophotometer. For cell dry weight (CDW) determination (w/v) algae samples (10 mL) were centrifuged (8,000 rpm; 5 min), washed twice with phosphate buffer, and dried at 60°C overnight until constant weight was attained. The chlorophyll “*a*” and “*b*” measurements were carried out by taking 10 mL of cell suspension. After separation (8,000 rpm; 5 min), the resulting pellet was added with acetone and ethanol in (1:1) ratio, and probe sonicator (Qsonica, Q55) was used for cell disruption (40 kHz; 2 min). The above steps were repeated twice, and the chlorophyll concentration was determined by measuring the OD of the supernatant at 647 nm and 664 nm for chlorophyll “*a*” and “*b*,” respectively, and was summed up for total chlorophyll using the following equation (Jeffrey and Humphrey, 1975).

(1) $$\begin{array}{rcl} \text{Chlorophyll }a\;\left(\mu \text{g}/\text{mL}\right) & = & \left(11.93\times {\text{A}}_{664}\right)-\left(1.93\times {\text{A}}_{647}\right) \end{array}$$

(2) $$\begin{array}{rcl} \text{Chlorophyll }b\;\left(\mu \text{g}/\text{mL}\right) & = & \left(20.36\times {\text{A}}_{647}\right)-\left(5.5\times {\text{A}}_{664}\right) \end{array}$$

Photosynthetic quantum yield (QY) was monitored by fluorescence measurements using PAM fluorometer (Aquapen, PSI). Substrate degradation in terms of COD and nitrate removal was estimated by standard procedures (APHA, 1998). Carbohydrate content was determined using the modified phenol-sulfuric acid method after acid hydrolysis of the algae biomass (Dubois et al., 1956), and the protein content was determined using Lowry method (Lowry et al., 1951). The total lipids were extracted by modified Bligh and Dyer method using chloroform and methanol (2:1) as solvents (Bligh and Dyer, 1959). The biomass pellet after separation (8,000 rpm; 10 min) was dried in hot air oven at 60°C for 24 h to obtain a constant weight. The dried biomass was ground into fine powder using mortar and pestle and subjected to alternating sonication (40 kHz, 2 min) and centrifugation (8,000 rpm, 10 min) thrice to extract maximum lipids from the algal biomass and pooled. Chloroform and methanol (2:1) were used for total lipids, whereas n-hexane was used for neutral lipids extraction (Miao and Wu, 2006). After centrifugation, the solvent: lipid layer was transferred into pre-weighed round bottom flask, and the total and neutral lipids were determined gravimetrically in terms of percentage dry cell weight (w/v).

### Fatty acid methyl esters (FAME) analysis

Oven dried biomass (100 mg) was subjected to methanol-sulfuric acid (2%) mixture and refluxed for 4 h. After the reaction time (4 h), the contents were rinsed with 25 mL of distilled water. The aqueous layer was extracted with ethyl acetate twice (2 mL × 25 mL). The extract was dried over anhydrous Na_2_SO_4_, concentrated under vacuum and used for analysis by GC-FID (Agilent 7680B) through packed column [Valcobond (VB) 30 m (0.25 mm × 0.25 mm)] using nitrogen as carrier gas (1 mL/min). The oven temperature was initially set to 140°C (for 5 min) and increased to 240°C at a ramping rate of 4°C/min for 10 min. FAME composition was compared with the standard FAME mix (C4-C24; SUPELCO).

### Nile red staining

Intracellular lipid bodies (LBs) in microalgae were observed with Nile red (9-diethylamino-5-benzo [α] phenoxazinone) staining (0.1 mg/mL; acetone; 100 μL suspension) (Subhash and Venkata Mohan, 2015). Prior to addition of the stain, the mixture of cells was washed with 2-mL phosphate-buffered saline (PBS) and 2-mL distilled water. Then, the cells were separated (5,000 rpm; 2 min) followed by 5 min incubation in the dark and pulse sonicated (2 secs; 40 kHz) for two to three times to allow the dye to pass through the cell wall. Fluorescent microscope (Nikon Eclipse 80i) with digital camera (YIM-smt, 5.5 megapixels) was used to capture cell images using Cy3 (excitation wavelength, 548 nm; emission wavelength, 561 nm and excitation laser lines 488 nm, 514 nm) and FITC (excitation wavelength, 495; emission wavelength, 519 nm and excitation laser lines 488 nm) fluorescent filters (NIS-elements D 3.0 software).

## Results and discussion

### Strain identification and phylogenetic analysis

**Figure 2:**
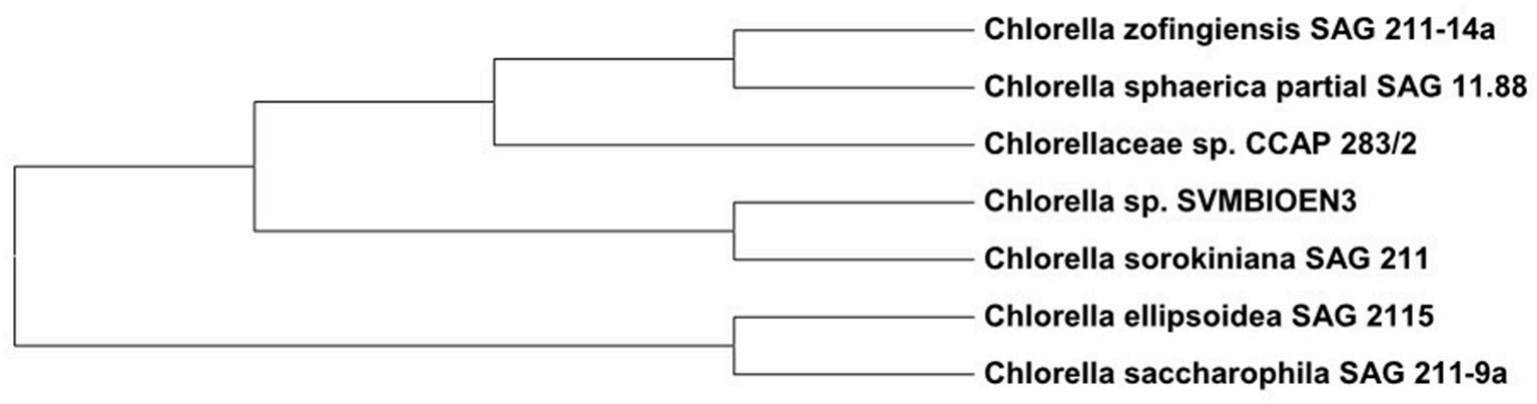
Phylogram showing the relationship of *Chlorella* sp. SVMBIOEN3 with other close relatives.

Based on the percentage similarities of ITS regions, the strain was identified as *Chlorella* sp. SVMBIOEN3 and the sequence was deposited with NCBI (Acc. No. KY350162). ITS gene sequence showed 99% similarity to *Chlorella sorokiniana* SAG811K. A phylogenetic tree showing the relationship among the evolutionary identical strains was constructed using the Molecular Evolutionary Genetics Analysis (MEGA) 6.0 software which is extensively used for high throughput molecular and evolutionary data anlaysis and phylogenetic tree construction (Zhang et al., 2018). The root node was fixed to differentiate between genus of *Chlorella* sp. and other genus (Micractinium) to clearly observe the evolutionary distance. Phylogenetic analysis based on ITS sequence analysis indicated that strain is closely related to *Chlorella sorokiniana*. The phylogenetic tree constructed based on neighborhood joining method has further confirmed that this strain is related to *Chlorella* sp. (Figure 2). ITS biotyping has been accepted as a universal tool for distinguishing eukaryotic species like microalgae and is also a good marker for molecular phylogenetic studies at lower taxonomic levels (González et al., 2001).

### Comparative assessment of nutritional modes

**Figure 3:**
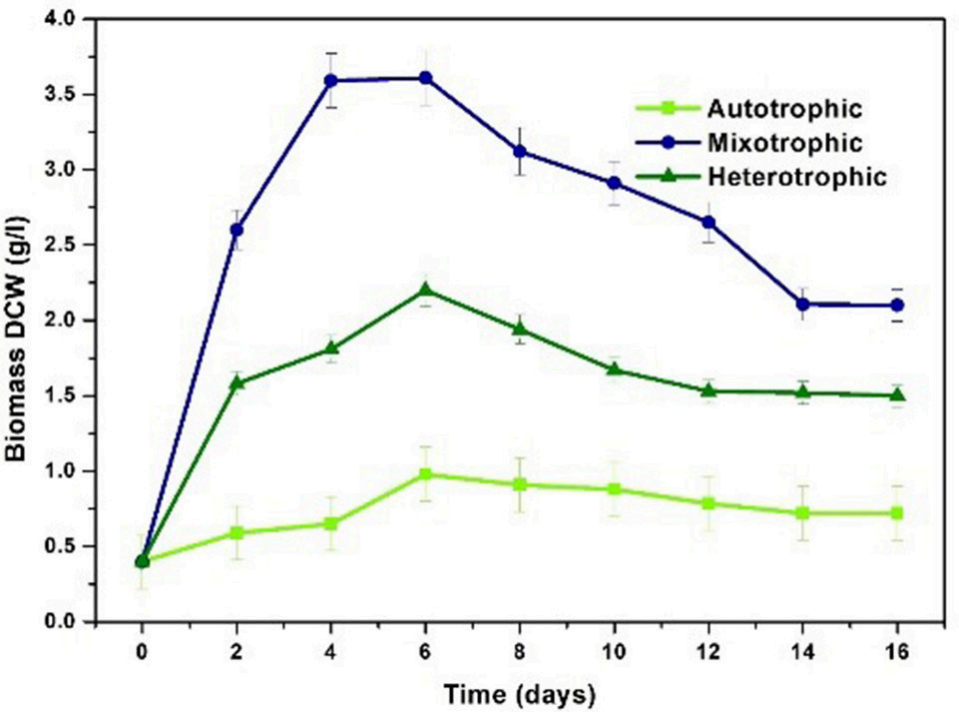
Biomass profile in three different nutritional modes.

*Chlorella* sp. was cultivated in three nutritional modes viz., autotrophic, mixotrophic, and heterotrophic. The experiments were carried out in two stages viz. growth phase (GP) and stress phase (SP). Maximum biomass growth was observed in mixotrophic mode at the end of 4th day of cultivation (3.59 g/L) followed by heterotrophic (1.58 g/L), and autotrophic (0.59 g/L) conditions within 6 days of cultivation (Figure 3). Biomass productivity also followed the similar trend where mixotrophic cultivation (448 mg/L/d) yielded higher values followed by heterotrophic (197 mg/L/d) and autotrophic (73.7 mg/L/d) conditions. During stress phase (SP), biomass showed constant decrement in all the three conditions reducing to 0.6 g/L, 2.1 g/L and 1.5 g/L in autotrophic, mixotrophic and heterotrophic conditions respectively.

Mixotrophic operation with 7.5 g/L glucose concentration showed maximum biomass concentration of 3.59 g/L and productivity of 448 mg/L/day compared to other nutritional modes under study. Similar results were observed by Wan et al. (2011) where maximum biomass content of 1.32 g/L was observed with *C. sorokiniana* when supplemented with 10 g/L of glucose under mixotrophic conditions. A maximum of 4.2 g/L biomass growth with *Chlorella vulgaris* NIES 227 strain using 12 g/L glucose concentrations was reported with mixotrophic operation (Shen et al., 2015). Mixotrophic cultivation requires glucose, CO_2_ and light in optimal proportions to gain the advantages of both CO_2_ and glucose as carbon source resulting in high density biomass. Glucose serves as simple and readily available source for production of ATP and energy carriers via glycolysis pathway. The subsequent by-products are utilized by algae cells for enhancing the biomass generating growth metabolites and thus increasing cell density (Michels et al., 2014; Rosenberg et al., 2014).

Autotrophic condition showed biomass content of 0.59 g/L with biomass productivity of 73.7 mg/L/d. Light intensity plays prominent role in autotrophic and mixotrophic conditions for enhancing growth of algal species. A light intensity of 56 μmol/m^2^/s with photo period of 12/12 h was employed in the present study. In a similar study, increase in specific growth rate and biomass productivity was observed with increase in light intensity from 37.5 to 65 μmol/m^2^/s (Khoeyi et al., 2012). Kim et al. (2013) evaluated *C. sorokiniana* cultivation under autotrophic, mixotrophic and heterotrophic conditions with light intensity of 60 μE/m^2^/s (Kim et al., 2013). Growth rate in mixotrophic conditions with light intensity of 33 W/m^2^ was higher while the growth rate in mixotrophic conditions with light intensity of 17 W/m^2^ was lesser than the heterotrophic conditions due to reduced light intensity (Chojnacka and Noworyta, 2004). Optimal light conditions are necessary for improving the biomass growth and lipid production.

Heterotrophic condition showed second highest biomass production (1.58 g/L) with a productivity of 197 mg/L/d. Relatively high amount of microalgal cell biomass (5.58 g/L) and lipid content (42%) was obtained in batch mode when *Chlorella* sp. was heterotrophically cultivated using a mixture of hydrolyzed molasses and de-oiled microalgal biomass residues (Zheng et al., 2015). Species like *Chlorella* are able to achieve very high cell densities during fed batch and batch cultivation. Heterotrophic cultivation is known to increase the biomass productivities and has the advantage of integration with low cost carbon sources like waste streams and elimination of light source (Doucha and Lívanský, 2006; Perez-Garcia et al., 2011; Venkata Mohan et al., 2015). Production of energy rich metabolites in heterotrophic cultivation is due to the oxidation of glucose via glycolysis pathway that results in redox carriers like ATP, NADPH, and other molecules yielding higher biomass productivity (Lu et al., 2017).

### Chlorophyll

**Figure 4:**
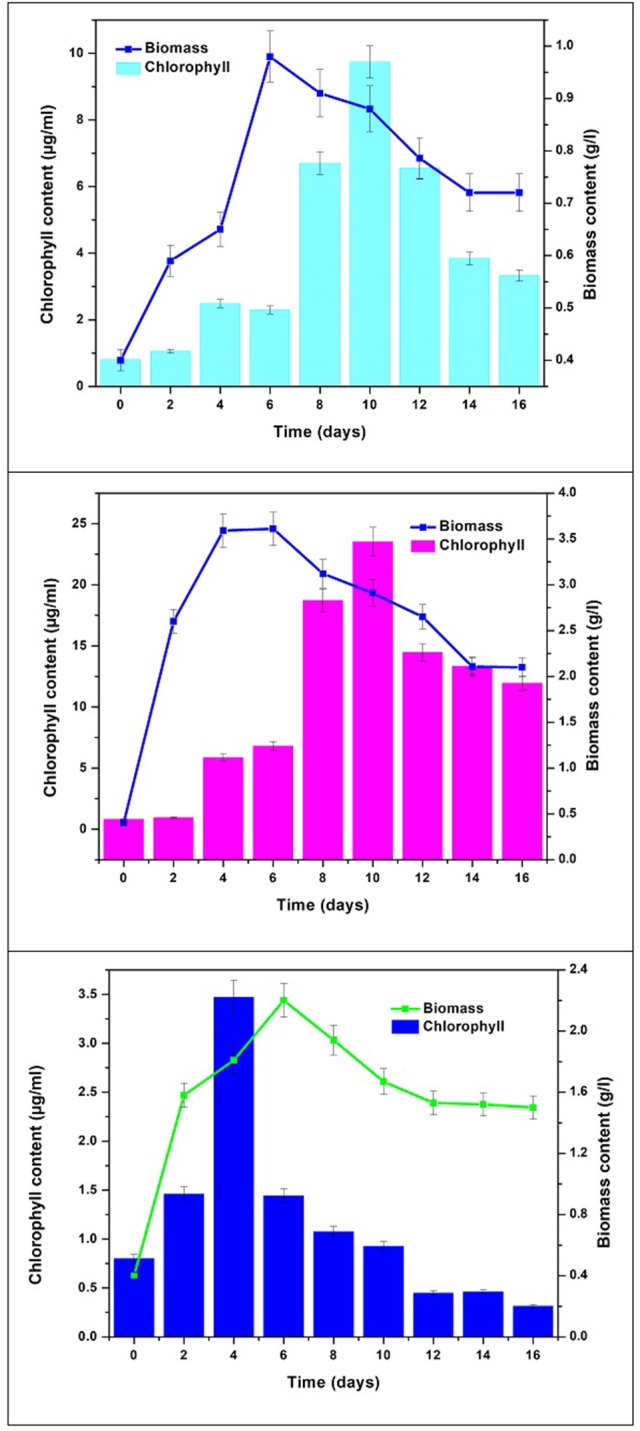
Biomass vs. Chlorophyll content in three nutritional modes.

Chlorophyll content was monitored to determine pigment modulation and understand the metabolite synthesis with changes in nutritional mode. Concentrations of chlorophyll “*a*” and “*b*” varied with respect to time in different modes of nutrition owing to their functional role in response to light. Chlorophyll “*a*” content was observed to be higher than chlorophyll “*b*.” Mixotrophic mode of cultivation showed higher total chlorophyll by the end of GP (23.51 μg/mL) followed by autotrophic mode (11.28 μg/mL) and the least was recorded in heterotrophic mode (4.2 μg/mL) (Figure 4). The maximum total chlorophyll in all the conditions reached maximum value at 10th day of operation. Mixotrophic conditions reported the highest chlorophyll *a* (16.53 μg/mL) and chlorophyll *b* (6.98 μg/mL) concentrations. In autotrophic mode, Chl *a* is observed to be higher (8.533 μg/mL) in comparison with Chl *b* (2.756 μg/mL). In heterotrophic mode, the concentrations of Chl *a* and Chl *b* were 2.648 and 1.553 μg/mL, respectively. Chlorophyll “*a*” content acts as a growth indicator and depicts the growth patterns in algal cultivation. All these pigments are naturally produced under phototrophic growth conditions. Considerable quantities of chlorophyll have also been detected under strictly heterotrophic conditions, indicating the constitutive expression of the photosynthetic system (Morales-Sánchez et al., 2013). Chlorophyll concentration can also be correlated with stress metabolism in algae cells. During stress phase (SP) chlorophyll pigments are usually degraded into simpler components under high levels of stress conditions, resulting in a considerable decrease in their content (Markou and Nerantzis, 2013; Subramanian et al., 2013). This may be the reason for gradual decrease in chlorophyll content during stress phase.

### Nutritional mode influence on lipid productivity

**Figure 5:**
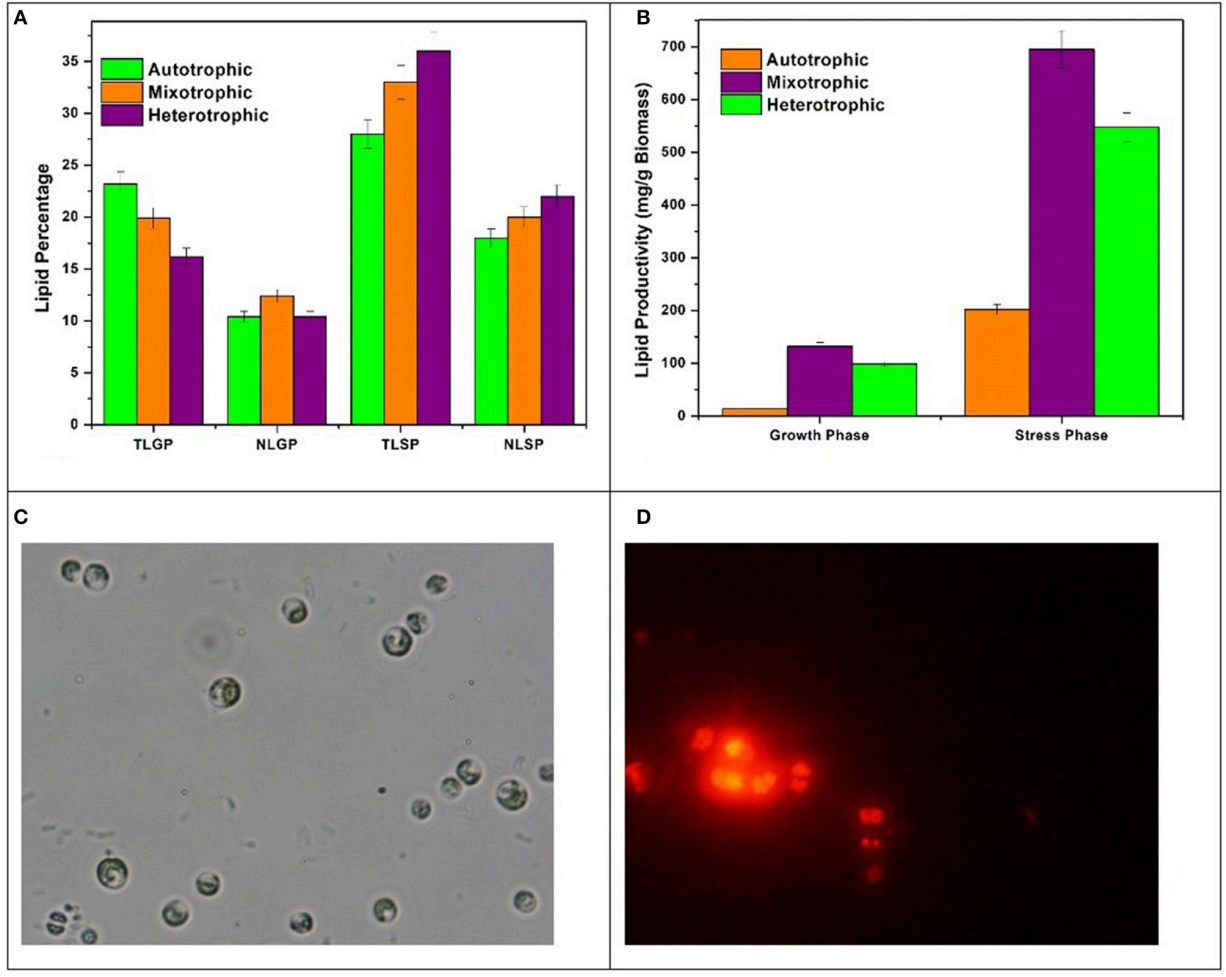
**(A)** Total and Neutral Lipid (TAG) percentages, **(B)** Lipid productivities in GP and SP, **(C)** Bright field, and **(D)** Nile Red staining of Chlorella sp. LBs in SP.

The lipid profile of *Chlorella* sp. cultivated in three nutritional modes varied with respect to growth phase (GP) and stress phase (SP). The total lipids (TL) obtained in GP are 23%, 19%, and 16% in autotrophic, mixotrophic and heterotrophic modes whereas neutral lipid (NL) content was 10%, 12%, and 9% respectively (Figure 5A). Influence of nutrient loss in stress phase (SP) was prominent with enhanced total and neutral lipid profiles. The TL from SP conditions obtained were 28%, 33%, and 36% whereas NL accumulated were 18%, 20%, and 22% in autotrophic, mixotrophic and heterotrophic conditions. Lipid productivities during GP were 8.2 mg/L/d, 3 mg/L/d and 0.9 mg/L/d in autotrophic, mixotrophic and heterotrophic conditions whereas productivities of 73.5 mg/L/d, 66 mg/L/d, and 22.5 mg/L/d were obtained at the end of SP. Based on the biomass content, the lipid productivity of biomass was also calculated for each condition. Biomass concentrations obtained were 13 mg/g, 132 mg/g, 98 mg/g in GP and 201 mg/g, 6,970 mg/g, and 547 mg/g after SP in autotrophic, mixotrophic and heterotrophic conditions (Figure 5B).

Different nutritional modes showed significant effect on the lipid synthesis with marked variations in the lipid profile. During GP, despite low biomass production, maximum TL percentage (23.2%) was obtained with autotrophic operation due to higher photosynthetic efficiency as can be observed by OJIP and NPQ fluorescence curves whereas NL were found higher in mixotrophic condition in GP. The influence of nutrient stress on neutral lipid/TAG formation in SP was also prominent. This may be due to reduction in biomass growth and activation of starch to lipid conversion pathways. During SP, heterotrophic dark condition has shown most influence on TL and NL production due to the presence of glucose as direct energy source for lipid synthesis and storage (Jia et al., 2015). The increase in the chlorophyll *b* content in mixotrophic condition is indicative of the shift toward neutral lipids and acts as a marker for high amounts of triacylglycerols (TAGs) (Pal et al., 2013). Bright field microscopy and Nile Red staining was performed at the end of SP to confirm lipid bodies (LBs) formation (Figure 5C). Distinguished LBs (Figure 5D) inside the microalgae cells in mixotrophic and heterotrophic conditions were observed. The activation of storage mechanism during stress phase might have resulted in significant forms of lipid accumulation in algae cells during SP.

#### Fatty acids profile

**Figure 6:**
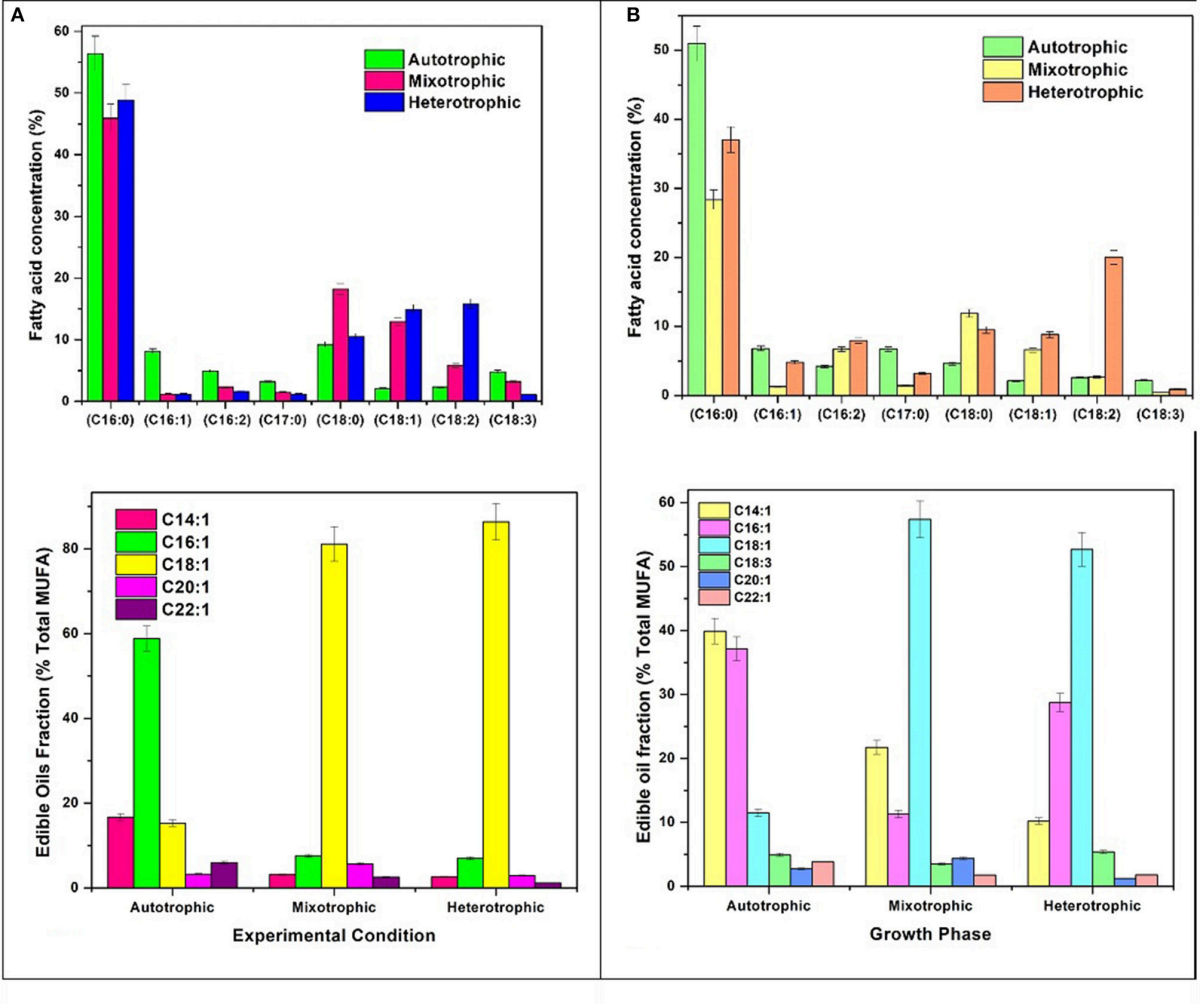
**(A)** Fatty acid composition and Edible fatty acids fraction in GP and **(B)** SP.

Varying concentrations of saturated (SFA) and unsaturated fatty acids (MUFA and PUFA) were detected in the (fatty acid methyl esters) FAME profile of all the three conditions (Table 1). The quantitative analysis of the FAME noticed presence of major fatty acids like C16:0, C16:1, C16:2, C17:0, C18:0, C18:1, C18:2, and C18:3 in all the nutritional modes of cultivation but with varying concentrations (Figure 6A). The monounsaturated fatty acids (MUFA) like C14:1, C16:1, C18:1, C20:1, C22:1 and polyunsaturated fatty acids (PUFA) like C16:2, C18:2, C18:3 were present in high fractions in mixotrophic and heterotrophic conditions whereas saturated fatty acid like C16:0 content was higher in autotrophic condition.

**Table 1:** Fatty acid composition of three nutritional modes.

**Fatty acid (% of Total FAME)**	**Autotrophic**	**Mixotrophic**	**Heterotrophic**
Caprylic acid (C8:0)	4.5	3.5	2.1
Lauric acid (C12:0)	1.1	0.4	0.3
Myristic acid (C14:0)	0.9	1.9	0.4
Myristoleic acid (C14:1)	7.3	2.5	1.7
Pentadecyclic acid (C15:0)	0.9	1.4	0.5
Palmitic acid (C16:0)	51	28.4	37.1
Palmitoleic acid (C16:1)	6.8	1.3	4.8
Hexadecadienoic acid (C16:2)	4.2	6.7	7.9
Margaric acid (C17:0)	6.7	1.4	3.2
Stearic acid (C18:0)	4.6	11.9	9.5
Oleic acid (C18:1)	2.1	6.6	8.8
Linoleic acid (C18:2)	2.6	2.7	20
Lineolenic acid (C18:3)	2.2	0.4	0.9
Arachidic acid (C20:0)	0.7	0.9	0.6
Paulinic acid (C20:1)	0.5	0.5	0.2
Eicosadecenoic acid (C20:2)	0.4	1	0.1
Erucic acid (C22:1)	0.7	0.2	0.3
Saturated fatty acid (SFA %) fraction	64.6	48.5	51
Polyunsaturated fatty acid (PUFA %)	18	36.6	33.2
Monounsaturated fatty acid (MUFA %)	17.4	14.9	15.8

##### Saturated fatty acids (SFA)

Palmitic acid (C16:0), a saturated fatty acid (SFA) was dominant in all three trophic conditions, but autotrophic mode yielded higher fraction (51%) followed by heterotrophic (37%) and mixotrophic (28.4%) conditions. Biodiesel favorable fatty acids majorly consisting of SFA (C16:0, C18:0) were prominent in heterotrophic condition with specific increment in linoleic acid (C18:2; 20%). Concentrations of palmitoleic acid (C16:2), stearic acid (C18:0), oleic acid (C18:1), and linoleic acid (C18:2) were high in mixotrophic condition which also favors biodiesel specifications (Fernandes et al., 2016). The autotrophic condition showed presence of biodiesel favorable fatty acids in high concentrations like palmitic (C16:0) and stearic acid (C18:0) and its derivatives in SP. Commercially important fatty acids like C16:0, C18:1, C18:2 were found to be increased in SP of heterotrophic condition. Mixotrophic operation showed higher concentrations of C16:0, C18:0, and C18:1. High concentrations of chlorophyll *a* in autotrophic mode facilitated high saturated fatty acid production, whereas higher concentrations of Chlorophyll *b* are associated with neutral lipids (Pal et al., 2013). SFA are known for good biodiesel properties as higher saturation can lead to better density and viscosity of biodiesel (Talebi et al., 2013). Also, SFA have greater calorific value due to presence of long chain hydrocarbons which release high energy (Chandra et al., 2014).

##### Polyunsaturated fatty acids (PUFA)

The fatty acid profiles showed marked variation with respect to unsaturation depending on the trophic mode of cultivation. In mixotrophic mode, higher concentrations of polyunsaturated fatty acids (PUFA; 16:2, C18:2, C18:3) were detected. Linolenic acid (C18:3) was found maximum in heterotrophic condition (5.3%) followed by autotrophic (4.9%) and mixotrophic (3.4%) operations. The higher concentrations of PUFA in mixotrophic and heterotrophic conditions may be due to the reorganization of membrane-based fatty acids from chloroplast to mitochondria and cytoplasmic lipid bodies (LBs) (Cohen and Khozin-Goldberg, 2005; Hu et al., 2008). The total PUFA content in mixotrophic and heterotrophic conditions were approximately 40% which can have good market potential in nutritional supplements. Heterotrophic dark condition may be favorable gamma linolenic acid or GLA (C18:3) synthesis and has relatively huge market in nutraceuticals and probiotics sector.

##### Monounsaturated fatty acids (MUFA)/edible oils fraction

Autotrophic operation yielded 17.4% of MUFA followed by heterotrophic (15.8%) and mixotrophic operation (14.9%) (Figure 6B). Myristoleic, palmitoleic, oleic, linolenic, paulinic, and erucic acids were predominantly observed in all conditions. Myristoleic (C14:1; 39.8% of MUFA) and palmitoleic (C16:1; 37.1%) acids were found to be dominant in autotrophic condition. Oleic acid (C18:1) content increased four folds in mixotrophic (57.3%) and heterotrophic (52.6%) cultivation. The major composition of TAG usually comprises of C16:0, C16:1, C18:0, C18:1, C18:2, and C18:3 which are desirable fatty acids for cooking oils from health perspective (Unkefer et al., 2017). Palmitoleic (C16:1) and oleic acids (C18:1) are known for their health benefits and thus are used as edible/cooking oils. Oleic acid is widely used in cooking oils due to mono unsaturation benefits to reduce low density lipoproteins (LDL) and helps in lowering blood pressure and related heart diseases (Terés et al., 2008).

### Quantum yield and PAM kinetics

Pulse amplitude modulation (PAM) provides instantaneous photosynthetic performance rates in microalgae and sensitively measures chlorophyll fluorescence parameters with respect to their adaptation to ambient environmental conditions. Quantum yield (QY), OJIP, and NPQ fluorescence kinetics were performed on *Chlorella* sp. SVMBIOEN3 to assess the changes in photosynthetic efficiencies with respect to trophic conditions. QY of autotrophic condition was observed to be 0.68, while 0.65 was obtained with mixotrophic condition and heterotrophic condition showed least at 0.63. The Fv/Fm range between 0.5 and 0.7 indicates that the algal cells are metabolically active and this was correlated with the growth phase where due to nutrient sufficient conditions, algal cells proliferate, and stay active (Young and Beardall, 2003; Lv et al., 2018). The incident light conversion efficiency is determined by quantum yield or Fv/Fm (variable/maximum fluorescence ratio) which is represented by the maximum photochemical quantum yield of PSII reaction centers. Fv/Fm parameter is extensively used as rapid fluorescence based photosynthetic performance indicator (Grossman and Takahashi, 2001; Yang et al., 2013).

**Figure 7:**
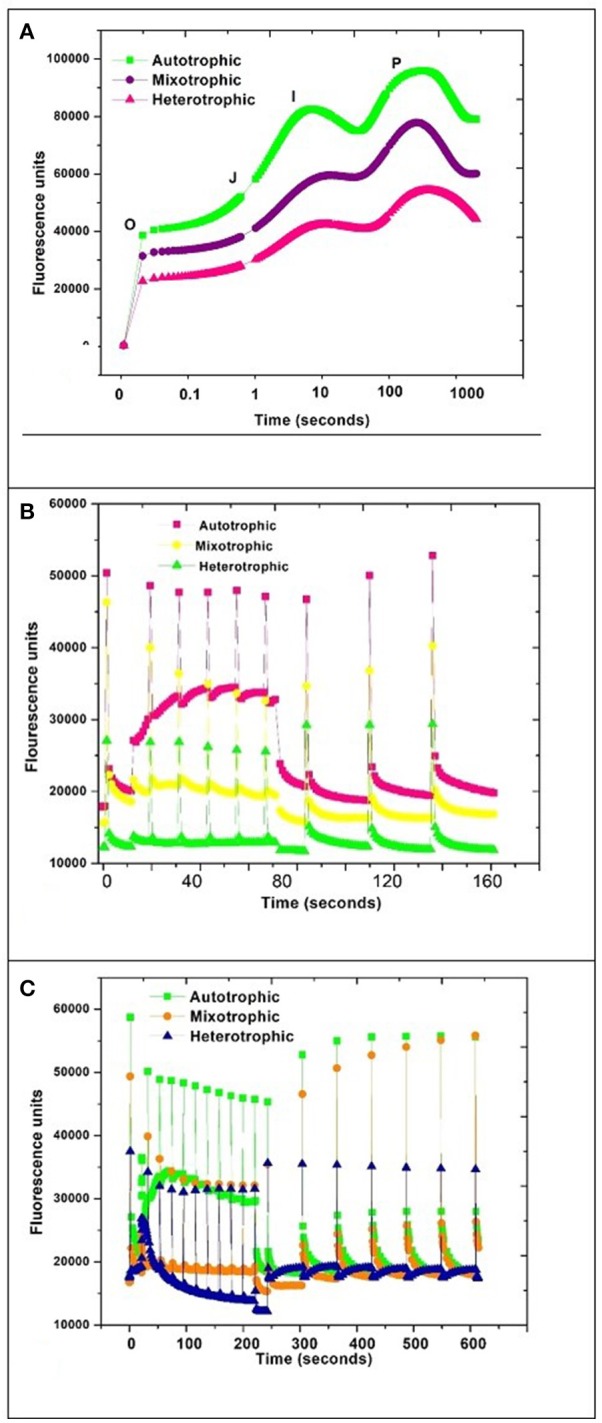
PAM kinetics of *Chlorella* sp. **(A)** OJIP, **(B)** NPQ1, and **(C)** NPQ2 during three nutritional modes.

Autotrophic condition showed maximal fluorescence activity followed by mixotrophic and heterotrophic condition in GP. The quantum yield was found to be directly proportional to photosynthetic activity. The OJIP curve showed decreasing trend with change in trophic condition (Figure 7A). The high photosynthetic activity was evident in autotrophic condition where the rubisco activity may be maximal due to photosynthetic mechanism as a major carbon fixation pathway.

A typical OJIP curve shows minimal fluorescence at inflection point O and gradually increases to inflection point J and maximal fluorescence I and drops to a final point P (Maxwell and Johnson, 2000; Fan et al., 2014). The steep depression and regaining of the fluorescence activity in autotrophic mode from the inflection point I to P can be correlated with the NPQ phenomenon due to stress initiation (Nikolaou and Bernardi, 2002; Solovchenko et al., 2013). NPQ1 was monitored for 160 s whereas NPQ2 was prolonged to 600 s. The outcome of a PAM protocol is a record of fluorescence characteristic fluxes:

(3) $$\begin{array}{rcl} \text{Quantum Yield}\left({\text{F}}_{\text{v}}/{\text{F}}_{\text{m}}\right) & = & \left({\text{F}}_{\text{m}}-{\text{F}}_{\text{o}}/{\text{F}}_{\text{m}}\right) \end{array}$$

(4) $$\begin{array}{rcl} \text{NPQ} & = & \left({\text{F}}_{\text{m}}-{\text{F}}_{\text{m}}^{\text{I}}\right)/{\text{F}}_{\text{m}}^{\text{I}} \end{array}$$

F = Dark fluorescence yield

F_o_ = Minimum fluorescence in a dark adapted sample

F_m_ = Maximum fluorescence in a dark adapted sample

${\text{F}}_{\text{o}}^{\text{I}}$ = Minimum fluorescence in a light adapted sample

${\text{F}}_{\text{m}}^{\text{I}}$ = Maximum fluorescence in a light adapted sample

F_v_/F_m_ = Maximum quantum efficiency of PSII (dimensionless)

A steep depression in curve was observed in the autotrophic condition and regained fluorescence between 10 and 100 s time window which may be attributed to NPQ phenomenon by activation of photo-protective mechanisms in algae (Muller et al., 2001; Figures 7B,C). The regaining of the fluorescence between inflection points I and P indicates active photo-protective mechanism in autotrophic mechanism due to the presence of photosynthetic pathways.

### Carbohydrate and protein

**Figure 8:**
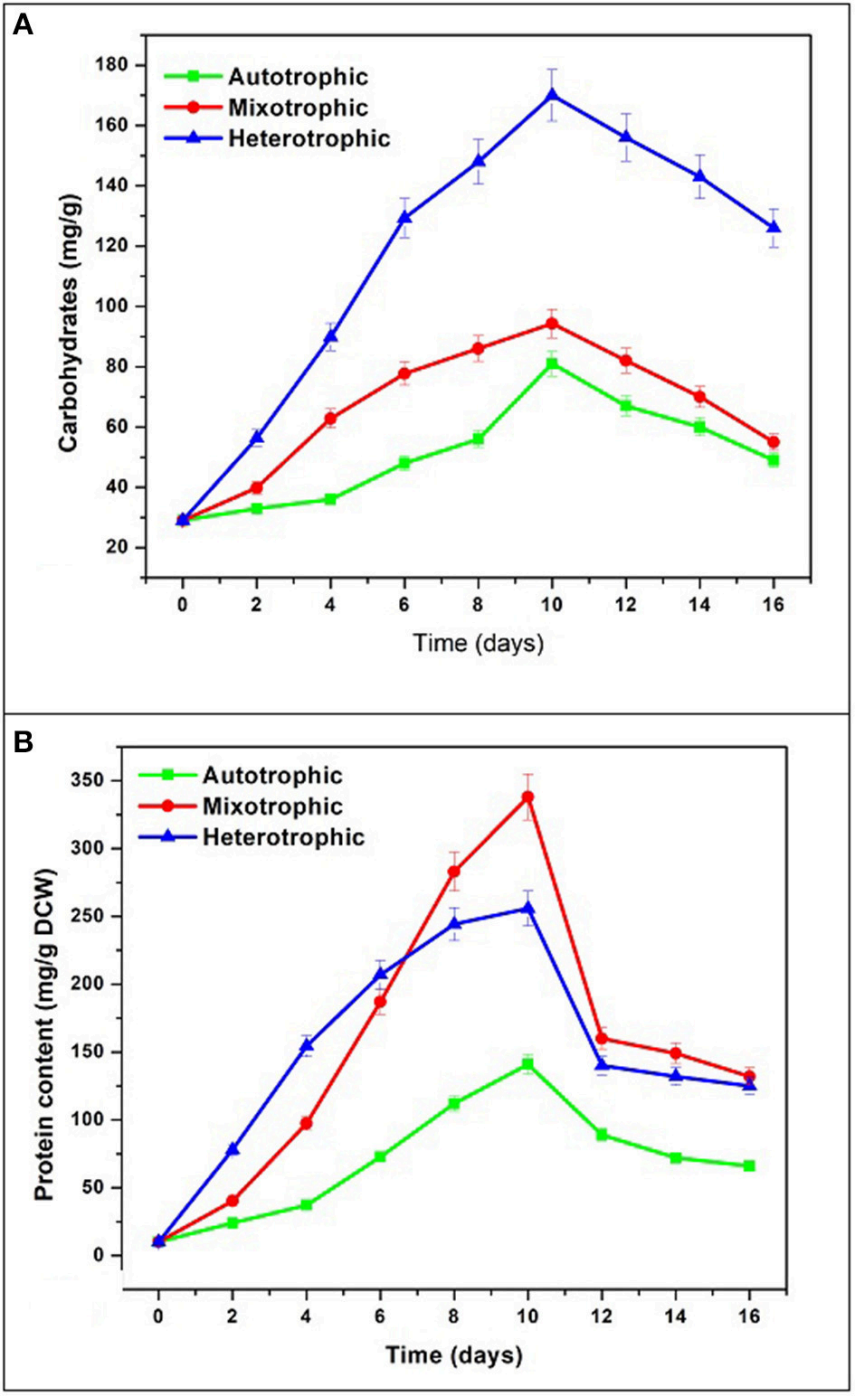
**(A)** Carbohydrate and **(B)** Protein content in three different nutritional modes.

The maximum accumulation of carbohydrates was observed in heterotrophic condition (170 mg/g) followed by mixotrophic (94.3 mg/g) and autotrophic (81 mg/g) conditions (Figure 8A). All conditions showed maximum accumulation of carbohydrates at end of 10 days followed by decreasing trend. During GP, the concentration of carbohydrates in heterotrophic condition (170 mg/g) was found approximately two times the maximum achieved in autotrophic mode (94 mg/g). In mixotrophic and heterotrophic conditions, glucose concentration was directly proportional to carbohydrate content. The effects of carbon sources (glucose and lactose) and cultivation modes (hetero- and mixotrophic) were investigated for the production of exopolysaccharides in *Neochloris oleoabundans* (Mata et al., 2014).

The protein content gradually increased and reached maximum in mixotrophic condition (338 mg/g) followed by heterotrophic condition (255 mg/g) and autotrophic mode (141 mg/g) (Figure 8B). In the present study, mixotrophic and heterotrophic conditions accumulated 40% w/w and 25% w/w protein content which has vast commercial applications in food industries. In the heterotrophic cultivation of the microalga *N. oleoabundans* with an equilibrated C/N ratio has been reported to allow protein accumulation (45% w/w). When no nutrient stress was present, proteins were the major macromolecules produced by algae in sufficient C/N ratio maintained during batch cultures (Markou and Nerantzis, 2013).

### COD and nitrate removal

**Figure 9:**
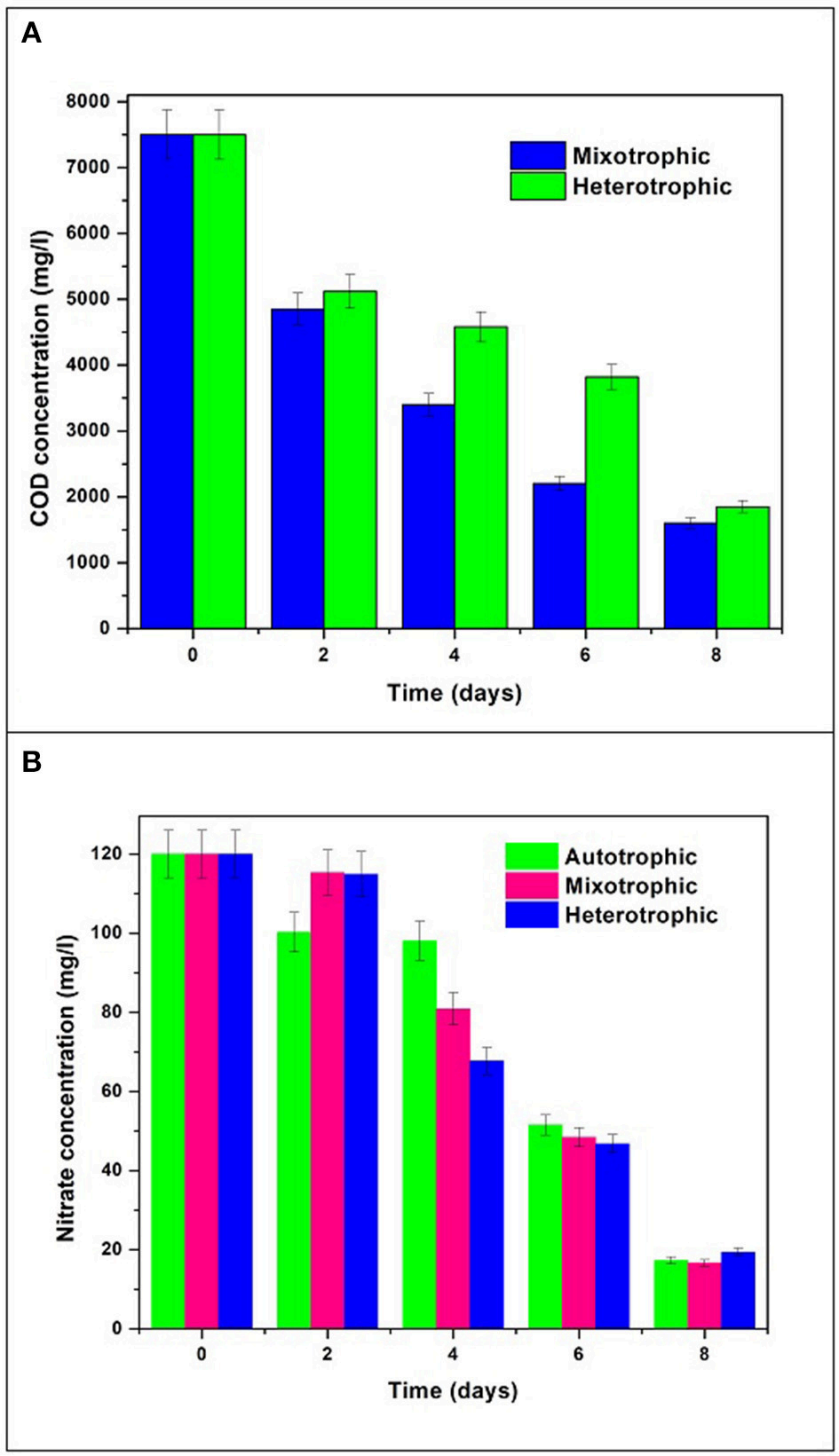
Nutrient removal in terms of **(A)** COD and **(B)** Nitrate removal.

The initial glucose concentration was 7.5 g/L COD which was reduced to 1.6 g/L with removal efficiency of 78% in mixotrophic mode and 1.85 g/L with removal efficiency of 75% in heterotrophic mode (Figure 9A). Initial concentration of nitrate in all the trophic modes was 120 mg/L which gradually reduced to 16 mg/L in autotrophic mode, 10 mg/L in mixotrophic mode, and 18 mg/L in heterotrophic mode by the end of the GP (Figure 9B). Nitrogen content plays important role in determining the chlorophyll concentration and thus related to growth mechanism in algae. The re-organization of chloroplast proteins and enzymes takes place when cells face low N availability during nutrient stress conditions (Evans and Seemann, 1989; Wu et al., 2011). This mechanism can be correlated with inversely proportional relationship of nitrate consumption and chlorophyll production in growth phase during the study.

## Conclusions

Mixotrophic and heterotrophic conditions yielded higher biomass (3.59 g/L and 1.9 g/L) during growth phase. Lipid content during stress phase increased from 15 to 35% in heterotrophic condition followed by 20 to 32% in mixotrophic condition. The lipid content was found to be higher in mixotrophic condition (670 mg/g biomass DCW) due to high biomass content (3.59 g/L) followed by heterotrophic condition (547 mg/g biomass DCW). Though biomass and lipid productivity was low in autotrophic condition, higher saturation (SFA) shows biodiesel favorable properties and can be performed in open pond raceway cultivation in an integrated biorefinery concept to favor economics. Mixotrophic and heterotrophic cultivations can be carried out in tubular/flat panel photobioreactors for synthesis of high value products. As the potential for microalgae as biodiesel feedstock diminishes, PUFA and edible oil production from neutral lipids is an attractive option toward nutraceutical and oil industries thus enabling bio-based economy.

## Author contributions

All authors listed have made a substantial, direct and intellectual contribution to the work, and approved it for publication.

### Conflict of interest statement

The authors declare that the research was conducted in the absence of any commercial or financial relationships that could be construed as a potential conflict of interest.
